# The anti-inflammatory potential of cefazolin as common gamma chain cytokine inhibitor

**DOI:** 10.1038/s41598-020-59798-3

**Published:** 2020-02-19

**Authors:** Barbara Żyżyńska-Granica, Bartosz Trzaskowski, Małgorzata Dutkiewicz, Oliwia Zegrocka-Stendel, Maja Machcińska, Katarzyna Bocian, Magdalena Kowalewska, Katarzyna Koziak

**Affiliations:** 1 Department of Pharmacodynamics, Centre for Preclinical Research and Technologies, Medical University of Warsaw, Banacha 1b, 02-097 Warsaw, Poland; 2 Chair and Department of Biochemistry, Medical University of Warsaw, Banacha 1, 02-097 Warsaw, Poland; 3 University of Warsaw, Centre of New Technologies, Banacha 2c, 02-097 Warsaw, Poland; 4 Department of Immunology, Biochemistry and Nutrition, Centre for Preclinical Research and Technologies, Medical University of Warsaw, Banacha 1b, 02-097 Warsaw, Poland; 5 Laboratory of Parasitology, Military Institute of Hygiene and Epidemiology, Kozielska 4, 01-163 Warsaw, Poland; 6 Department of Immunology, Faculty of Biology, University of Warsaw, Miecznikowa 1, 02-096 Warsaw, Poland; 7 Department of Molecular and Translational Oncology, Maria Sklodowska-Curie Institute - Oncology Centre, Roentgena 5, 02-781 Warsaw, Poland

**Keywords:** Pharmaceutics, Psoriasis

## Abstract

A continuing quest for specific inhibitors of proinflammatory cytokines brings promise for effective therapies designed for inflammatory and autoimmune disorders. Cefazolin, a safe, first-generation cephalosporin antibiotic, has been recently shown to specifically interact with interleukin 15 (IL-15) receptor subunit α (IL-15Rα) and to inhibit IL-15-dependent TNF-α and IL-17 synthesis. The aim of this study was to elucidate cefazolin activity against IL-2, IL-4, IL-15 and IL-21, i.e. four cytokines sharing the common cytokine receptor γ chain (γ_c_). *In silico*, molecular docking unveiled two potential cefazolin binding sites within the IL-2/IL-15Rβ subunit and two within the γ_c_ subunit. *In vitro*, cefazolin decreased proliferation of PBMC (peripheral blood mononuclear cells) following IL-2, IL-4 and IL-15 stimulation, reduced production of IFN-γ, IL-17 and TNF-α in IL-2- and IL-15-treated PBMC and in IL-15 stimulated natural killer (NK) cells, attenuated IL-4-dependent expression of CD11c in monocyte-derived dendritic cells and suppressed phosphorylation of JAK3 in response to IL-2 and IL-15 in PBMC, to IL-4 in TF-1 (erythroleukemic cell line) and to IL-21 in NK-92 (NK cell line). The results of the study suggest that cefazolin may exert inhibitory activity against all of the γ_c_ receptor-dependent cytokines, i.e. IL-2, IL-4, IL-7, IL-9, IL-15 and IL-21.

## Introduction

Inflammation resulting from excess pro-inflammatory cytokine production, aberrant cytokine responsiveness or deficient anti-inflammatory response is recognized as a major contributor to *i.a*. atherosclerosis, cancer, autoimmunity, and an important factor in chronic infections and age-related conditions (inflammaging)^1,2^. Clinical introduction of biological drugs targeting TNF-α, IFNα/β, IFN-γ, IL-1, IL-2, IL-5, IL-17, IL-18 and IL-22 fundamentally changed the therapeutic algorithms and outcomes of numerous, clinically distinct disorders^3,4^. Thus, efficient suppression of cytokines, their receptors or their downstream signal transduction pathways providing suppression of chronic immune activation further validated the concept of monoclonal antibody and small-molecule inhibitor-based therapeutics as anti-inflammatory drugs.

Extensive exploration of inhibitors of protein-protein interactions targeting inflammatory signals has recently led to the discovery of cefazolin as an antagonist of α chain of IL-15 receptor (IL-15Rα, CD215) and effective inhibitor of IL-15 biological activity^5^. As a first-generation cephalosporin antibiotic, cefazolin is used worldwide (Ancef® and Kefzol® in the US and Canada, Kefzol® in the UK, Gramaxin® and Elzogran® in Germany, Cefamezin® in Holland and Japan, Cefacidal® in France, and Cefazolin Sandoz or Tarfazolin in Poland) since the early 1970s and has well-documented pharmacokinetic, toxicology and safety records. Thus, repositioning of the drug to another therapeutic area could benefit from the advantage of decreased development costs and decreased time to market.

The inhibition of IL-15 activity by cefazolin *via* IL-15Rα does not exclude the possibility of additional mechanisms underlying the drug action. IL-15 binds to its receptor which consists of three different subunits with specific functions: the α chain confers ligand specificity, the common gamma chain (γ_c_, CD132) is necessary for signal transduction, while the β subunit (IL-2/IL-15Rβ, CD122) participates in both events. In a unique IL-15 signalling mechanism two associations with its receptor are possible. In *cis*-presentation the IL-15∙IL-15Rα complex requires IL-15Rβγ_c_ assembled on the same cell while in *trans*-presentation the IL-15∙IL-15Rα complex is presented to other cells, which express IL-2/IL-15Rβγ_c_ (or possibly also IL-2Rαβγ_c_ or/and IL-15Rαβγ_c_) in their cell membranes. Regulating the function of immune cells through *trans*-presentation seems to be the prevailing mechanism of IL-15 action^6,7^. It could be therefore assumed that cefazolin inhibitory efficacy against IL-15 may originate not only from IL-15Rα inhibition, but also from IL-2/IL-15Rβ or/and γ_c_ suppression. If confirmed, this could provide a compelling rationale for the use of cefazolin as an inhibitor of all cytokines binding to γ_c_, *i.e*. IL-2, IL-4, IL-7, IL-9, IL-15, and IL-21^8^.

To test the hypothesis that cefazolin is not only an antagonist of IL-15Rα, but it also interferes with ligand binding to IL-2/IL-15Rβ and γ_c_ we evaluated the drug activity against four cytokines of the common γ_c_ family: IL-2, IL-4, IL-15 and IL-21, in different cell types *in vitro*. Each of these cytokines acts through differently assembled receptors: IL-2 and IL-15 through heterotrimeric receptor complexes consisting of IL-2Rα or IL-15Rα, IL-2/IL-15Rβ and γ_c_, while IL-4 and IL-21 through heterodimeric IL-4Rαγ_c_ and IL-21αγ_c_, respectively. *In silico* approach was used to identify cefazolin-IL-2/IL-15Rβ and γ_c_ receptor binding mode.

## Results

### IL-2/IL-15Rβ and γ_c_ contain potential binding sites for cefazolin

**Figure 1:**
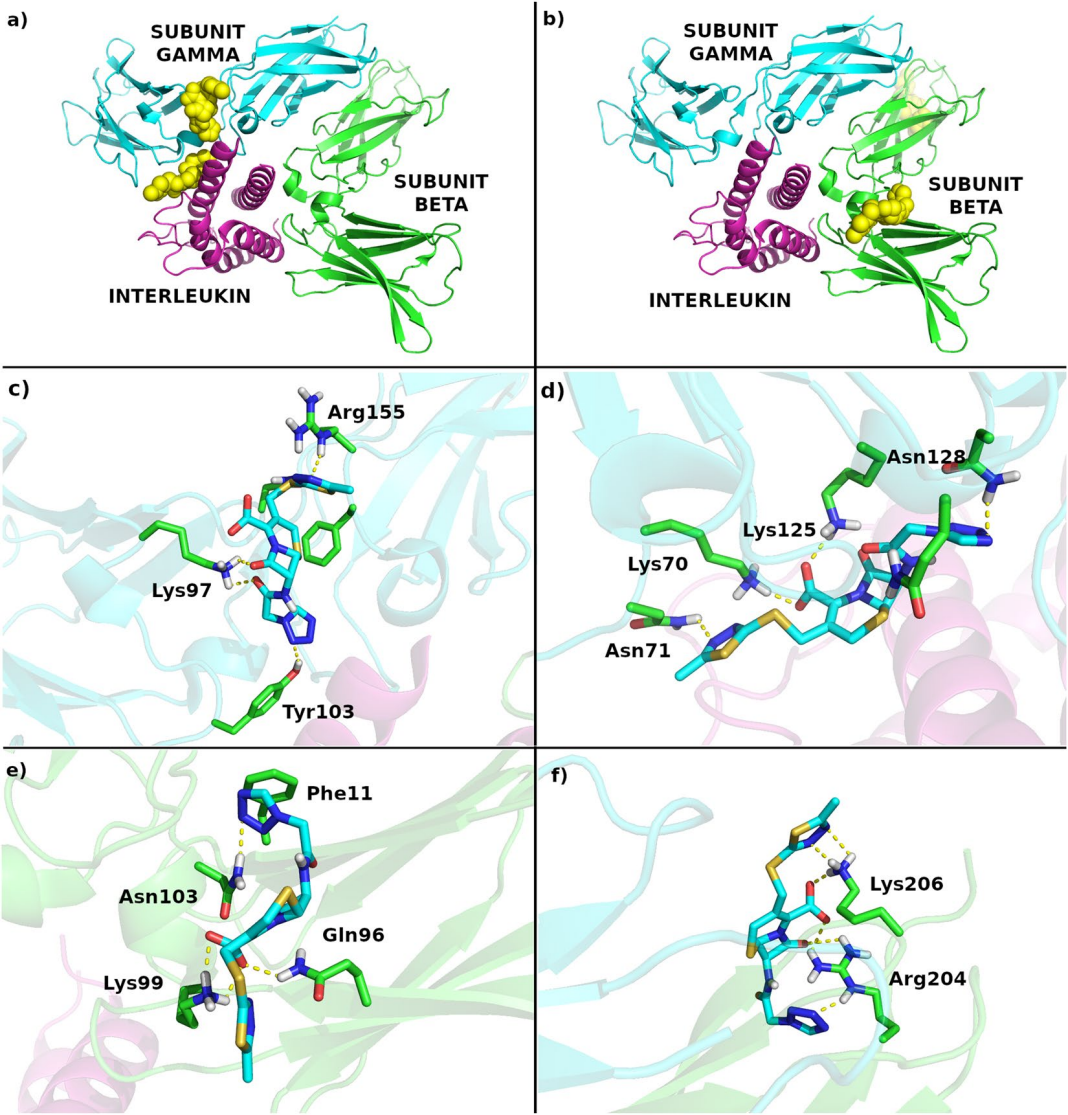
Potential cefazolin binding sites in IL-2/IL-15Rβ and γ_c_. (**a**) Potential cefazolin binding sites within γ_c_; (**b)** potential cefazolin binding sites within IL-2/IL-15Rβ; (**c**) the first potential cefazolin binding site in γ_c_; (**d**) the second potential cefazolin binding site in γ_c_; (**e**) the first potential cefazolin binding site in IL-2/IL-15Rβ; (**f**) the second potential cefazolin binding site in IL-2/IL-15Rβ.

The hypothesis that cefazolin binds to IL-2/IL-15Rβ and/or γ_c_ was first verified *in silico*. Two potential binding sites of very high binding affinities for cefazolin were revealed by molecular docking in each of the receptor subunits (K_i_ of 72/87 pM and 70/79 pM for IL2/IL-15Rβ and γ_c_, respectively). These values are lower than our previously reported estimates of cefazolin binding affinity to IL-15Rα which suggests that the drug binds preferentially to IL-2/IL-15Rβ and/or γ_c_^5^. Both of the cefazolin binding sites in IL-2/IL-15Rβ chain are located relatively far from the γ_c_ and the cytokine. In the first potential binding site (K_i_ = 72 pM) cefazolin interacts with K99 (salt bridge), Q96 and N103 (hydrogen bonds) as well as with F11 and F191 (hydrophobic and van der Waals interactions) (Fig. 1). In the second potential binding site (K_i_ = 87 pM) cefazolin is stabilized in the binding site mainly by two salt bridges with R204 and K206. Despite the fact, that the binding sites are relatively far from both IL-2/IL-15Rβ-γ_c_ and IL-2/IL-15Rβ-cytokine interfaces, it could be speculated that upon attachment cefazolin alters the dynamic structure of the entire heterotrimeric receptor complex, which may lead to the disruption of the cytokine-receptor binding interaction. In γ_c_, both of the potential cefazolin binding sites are located close to the γ_c_-cytokine interface (Fig. 1). In the first potential binding site cefazolin is stabilized by three strong interactions: the hydrogen bond between Tyr103 and tetrazole part of the drug, Lys97 salt bridge to carboxylic acid of cefazolin and Arg155 interaction with the thiadiazole moiety of the drug. In the second potential binding site cefazolin interacts with Asn128 (tetrazole part), Lys125 and Lys70 (carboxylic acid) and Asn141 (thiadiazole part). It could be hypothesized that in the presence of cefazolin the cytokine binding to the γ_c_ is partially disrupted, either *via* direct formation of the γ_c_-cefazolin complex or by structural changes to the γ_c_ imposed by the presence of the drug. Since the binding affinities of cefazolin to IL-2/IL-15Rβ and γ_c_ subunits are similar and within the expected accuracy of the docking algorithm, we have not been able to unequivocally determine the binding mode of cefazolin, though its slightly higher binding affinity to γ_c_ may indicate preferential binding to this receptor subunit.

### Cefazolin inhibits IL-2-, IL-4- and IL-15-induced cell proliferation

**Figure 2:**
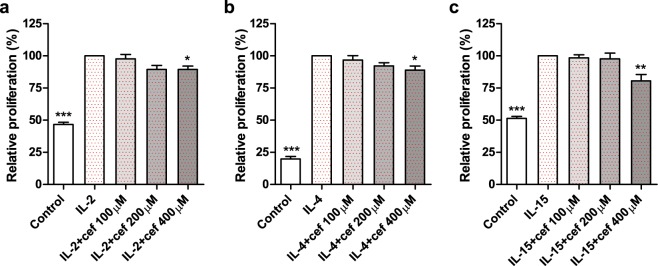
The influence of cefazolin on IL-2- (**a**), IL-4- (**b**) and IL-15-induced (**c**) cell proliferation measured by BrdU Cell Proliferation Assay. The results are presented as the percentage of PBMC response compared to IL-2- (**a**) and IL-15- (**c**) treated cells that were defined as 100% and TF-1 response compared to IL-4- (**b**) treated cells that were defined as 100%. Control refers to unstimulated cells. The results from three independent experiments (n = 3) are presented as mean ± SD. Statistical significance was assessed by ANOVA with Dunnet post hoc test. *p < 0.05, **p < 0.01, ***p < 0.001.

The effect of cefazolin in cells responding to cytokines *via* differently assembled IL-2/IL-15Rβ and/or γ_c_ was examined *in vitro*. The anti-proliferative effect of cefazolin was measured in two cell types: peripheral blood mononuclear cells (PBMC) and TF-1 erythroleukemic cells. Prior to the experiments, cefazolin cytotoxicity was tested in 100, 200, 400 and 800 µM concentrations, all of which were found nontoxic (data not shown). PBMC divide after stimulation with IL-2 and IL-15^9,10^, while TF-1 cells proliferate following exposure to IL-4^11^. The analysis of DNA synthesis using BrdU Cell Proliferation Assay revealed a reduction of cell response to IL-2 (20 ng/ml; 420 U/ml), IL-15 (5 ng/ml; 2,250 U/ml) or IL-4 (10 ng/ml; 290 U/ml) stimulation in presence of 400 µM cefazolin (Fig. 2). We confirmed these observations by assessing cefazolin influence in phytohaemagglutinin (PHA)-activated PBMC using CellTrace™ CFSE Cell Proliferation Kit. PHA is a non-specific T-cell activator known to potently stimulate lymphocyte mitotic activity and cytokine production. The data showing inhibitory effect of 200 µM and 400 µM cefazolin on IL-2 and IL-15-dependent proliferation of PHA-activated PBMC, respectively, are presented in Figure S1 (Supplementary Material). Despite the simplicity of the PHA model these results give some additional insight into cefazolin action in conditions which are immunologically more robust. To validate results for IL-4, we performed mitochondrial activity (MTT) assay, which revealed stronger inhibitory effect of cefazolin and reached statistical significance already at 100 µM concentration (Figure S2, Supplementary Materials). Since for most cell populations mitochondrial activity is related to the number of viable cells, the assay is broadly used to measure cell proliferation. However, MTT test is also used to quantify cell activity, independently of proliferation^12^. Thus, stronger response to cefazolin detected in MTT assay may reflect the combination of the drug effect on cell proliferation (more sensitively measured with BrdU) and on other cellular processes.

Reduction in IL-2, IL-4 and IL-15-induced cell proliferation caused by cefazolin, although not robust, suggests that the drug interferes not only with IL-15Rα, as reported previously^5^, but also with IL-2/IL-15Rβ and/or γ_c_.

### Cefazolin inhibits IL-2- and IL-15-induced IFN-γ, IL-17 and TNF-α production

**Figure 3:**
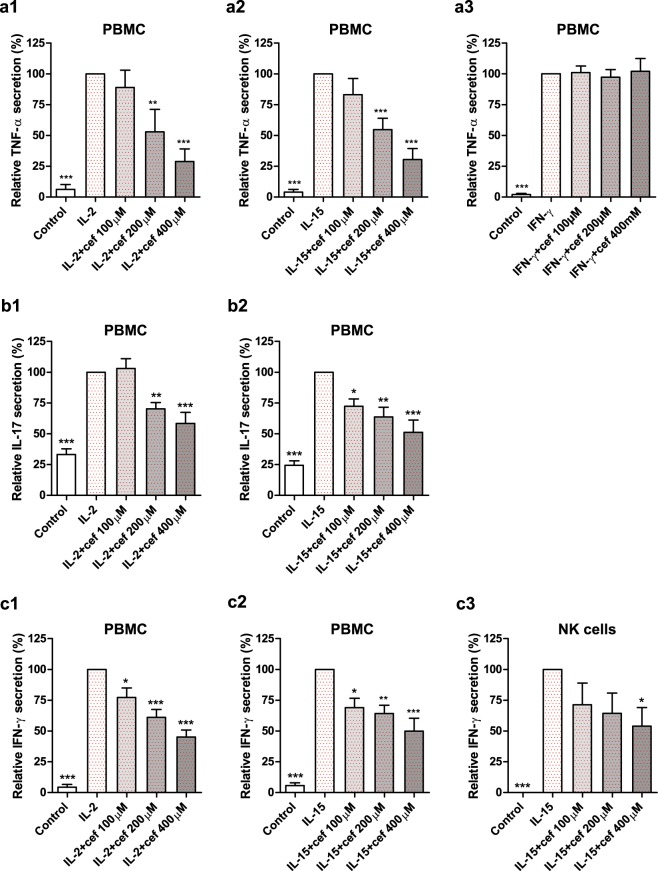
The influence of cefazolin on TNF-α, IL-17 and IFN-γ release. TNF-α secretion in IL-2 (**a**1), IL-15 (**a**2) and IFN-γ (**a**3) -stimulated PBMC; IL-17 secretion in IL-2 (**b**1) and IL-15 (**b**2) stimulated PBMC; (**c**) IFN-γ secretion in IL-2 (**c**1) and IL-15 (**c**2) stimulated PBMC as well as IL-15-stimulated NK cells (**c**3). The cytokine secretion was donor-dependent and for TNF-α varied from 114.2 to 1,451.4 pg/mg of total protein for IL-2-stimulated PBMC (**a**1), from 98.5 to 1,013.5 pg/mg of total protein for IL-15-stimulated PBMC (**a**2) and from 27.9 to 1,712.8 pg/mg of total protein for IFN-γ-stimulated PBMC (**a**3); IL-17 secretion varied from 35.4 to 921.5 pg/mg of total protein for IL-2 stimulated PBMC (**b**1) and from 36.6to 1,442.1 pg/mg of total protein for IL-15 stimulated PBMC (**b**2); IFN-γ secretion varied from 55.06 to 4,463.4 pg/mg of total protein for IL-2 stimulated PBMC (**c**1); from 10.79 to 3141.4 pg/mg of total protein for IL-15-stimulated PBMC (**c**2) and from 791.7 to 2174.6 pg/mg of total protein for IL-15-stimulated NK cells (**c**3). The results are presented as relative cytokine secretion (%) compared to IL-2- (**a**1, **b**1, **c**1), IL-15- (**a**2, **b**2, **c**2, **c**3) or IFN-γ- (**a**3) stimulated cells that were defined as 100%. Control refers to unstimulated cells. The results from at least three independent experiments (n = 3–5) are presented as mean ± SD. Single donor data are presented in Supplementary Figures S3, S4 and S5. Statistical significance was assessed by ANOVA with Dunnet post hoc test. *p < 0.05, **p < 0.01, ***p < 0.001.

Synthesis of numerous pro-inflammatory cytokines, including IL-17, IFN-γ and TNF-α, is one of the hallmark responses of human lymphocytes to IL-2 and IL-15^13,14^. Therefore, blocking of IL-2/IL-15Rβ and/or γ_c_ subunits should suppress the cellular production of cytokines in response to IL-2 or IL-15 stimulus. As shown in Fig. 3 and in Supplementary Figures S3, S4 and S5, the obtained results revealed the concentration-dependent inhibitory effect of cefazolin on IL-17, IFN-γ and TNF-α synthesis assessed in PBMC and NK cells stimulated with IL-2 or IL-15. A decrease in IL-17 production was significant at 100 µM and 200 µM concentrations of the drug in IL-15 (5 ng/ml; 2,250 U/ml) and IL-2 (20 ng/ml; 420 U/ml) stimulated PBMC, respectively. Diminished secretion of IFN-γ by IL-2 and IL-15-stimulated PBMC was observed at 100 µM cefazolin. In IL-15-stimulated NK cells this effect was statistically significant at 400 µM concentration of the drug.

To verify the specificity of the observed cefazolin-dependent inhibition of cytokine synthesis in cells stimulated with IL-2 and IL-15 – common γ_c_ cytokine family members, we assessed the drug effect on TNF-α synthesis in PBMC stimulated with IFN-γ (5 ng/ml; 100 U/ml). IFN-γ is a pro-inflammatory cytokine which acts through a heterodimeric IFNγR complex consisting of IFNγR1 and IFNγR2 and does not bind neither to IL-15Rα, IL-2/IL-15Rβ nor to γ_c_ receptor subunit^15^. Thus, a specific inhibitor of IL-2/IL-15Rβ and/or γ_c,_ should not interfere with IFNγR and its downstream signalling. As shown in Fig. 3, cefazolin did not affect IFN-γ-dependent TNF-α production in PBMC while it strongly inhibited TNF-α secretion induced by IL-2- and IL-15-stimulation. The effect was observed at 200 µM concentration of the drug and was concentration-dependent. The presented results further strengthen the hypothesis that cefazolin is an inhibitor of IL-2/IL-15Rβ and/or γ_c_.

### Cefazolin reduces surface expression of CD11c in monocyte-derived dendritic cells

Dendritic cells (DC) phagocyte, process and present antigen to lymphocytes, thus play a crucial role in initiating and directing cellular and humoral immune responses. DC can be generated *in vitro* from blood monocytes cultured in presence of IL-4 and GM-CSF^16^. The resulting monocyte-derived DC highly express on their cell surface a transmembrane integrin alpha X also known as CD11c, a classical marker of DC. This molecule is of critical importance in the process of efficient antigen uptake by phagocytosis and transition of DC from antigen processing to antigen-presenting cells.

**Figure 4:**
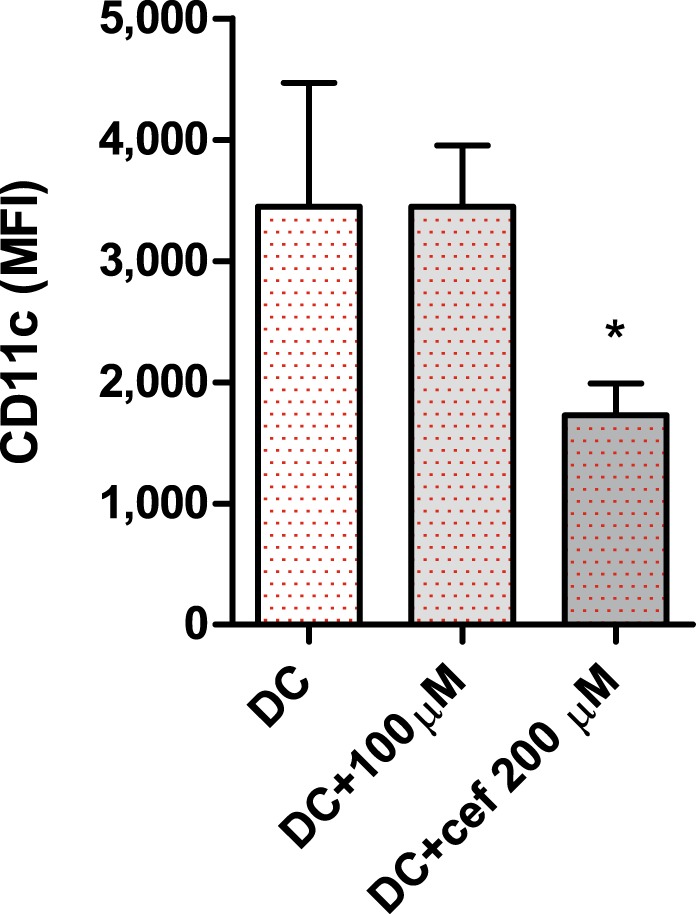
The effect of cefazolin on surface CD11c expression in monocyte-derived DC. DC were generated *in vitro* from human monocytes cultured in presence of IL-4 and GM-CSF with or without cefazolin. Surface CD11c expression in CD14- cells treated with IL-4 and GM-CSF was assessed using CD11c-APC and CD14-PE monoclonal antibodies and flow cytometry method. The results are presented as mean ± SD of mean fluorescent intensity (MFI) from three independent experiments (n = 3) with cells obtained from different donors.

As shown in Fig. 4, 200 μM cefazolin significantly decreased surface expression of CD11c in monocyte-derived DC harvested on day 5 of culture. This finding suggests that cefazolin impairs DC differentiation and function most probably by affecting IL-4-dependent processes. Higher concentrations of cefazolin (400 μM) decreased cell viability (data not shown) therefore were not used in the experiments.

### Cefazolin inhibits IL-2, IL-4, IL-15 and IL-21-stimulated JAK3 phosphorylation

**Figure 5:**
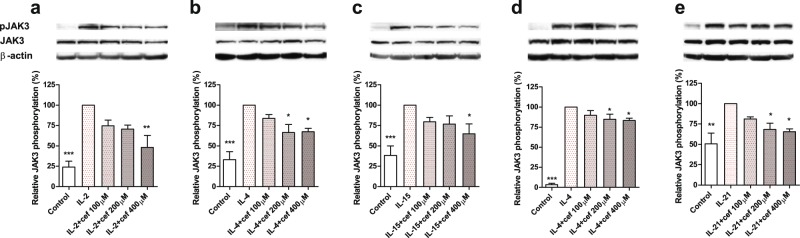
Cefazolin effect on JAK3 phosphorylation. Representative western blots with accompanying densitometry of at least three experiments are shown for phospho-JAK3 (pJAK3), JAK3 (JAK3) and β-actin in cell lysates obtained after cytokine and cefazolin treatment: (**a**) PBMC stimulated with IL-2; (**b**) PBMC stimulated with IL-4; (**c**) PBMC stimulated with IL-15; (**d**) TF-1 cells stimulated with IL-4; (**e**) NK-92 cells stimulated with IL-21. Volume of bands was calculated by the means of Image Lab 5.2 Software (BioRad). JAK3 phosphorylation was quantified as the phospho-JAK3/JAK3 ratio and is presented as the percentage of cell response relative to IL-2- (**a**), IL-4- (**b**, **d**), IL-15- (**c**) or IL-21- (**e**) treated cells (100%). Control refers to unstimulated cells. The results are presented as mean ± SD from three independent experiments (n = 3). Statistical significance was assessed by ANOVA with Dunnet post hoc test. *p < 0.05, **p < 0.01, ***p < 0.001. Full-length blots are presented in Supplementary Figure S6. Single donor data are presented in Supplementary Figures S7–S11. Statistical significance was assessed by ANOVA with Dunnet post hoc test. *p < 0.05.

Janus kinase (JAK)-family protein tyrosine kinases are physically associated with cytokine receptors. JAK3 is constitutively associated with γ_c_ and upon phosphorylation triggered with a cytokine it activates JAK1, the major player in γ_c_ cytokine signaling. We found that phosphorylation of JAK3 in response to the cytokine treatment is significantly diminished after cefazolin treatment. This effect was observed at 200–400 µM concentrations of the drug in western blotting analyses of IL-2-, IL-4- and IL-15-treated PBMC, IL-4-stimulated TF-1 and IL-21-treated NK-92 cells (Fig. 5 and Supplementary Figures S6–S11). It may be therefore concluded that cefazolin suppresses signal transduction by γ_c_ receptors.

## Discussion

Advances in crystallography and *in silico* techniques provide promising opportunities in the design of protein-protein interaction inhibitors for therapeutic purposes^17^. Detailed information about the 3D structure and molecular determinants of the specific, high affinity interaction between IL-15 and IL-15Rα^18,19^, combined with docking and experimental methods resulted in our recent discovery of cefazolin – an old, safe first-generation cephalosporin antibiotic, as promising IL-15Rα inhibitor^5^. In the present study we used broader approach to determine cefazolin potential to interfere with the two other IL-15R subunits: the IL-2/IL-15Rβ, which is shared with IL-2, and the γ_c_ – a common component of receptors for IL-2, IL-4, IL-7, IL-9, IL-15 and IL-21. Molecular docking unveiled two potential cefazolin binding sites within IL-2/IL-15Rβ and two within γ_c_ subunit, all with estimated K_i_ values in the 70–90 pM range.

It has been generally acknowledged that although the protocol used in this study (and other available docking protocols) is accurate in predicting ligand poses within binding sites of various receptors, it is inaccurate in estimating binding free energies, which are often different by a few orders of magnitude^20,21^. Therefore while the obtained ligand poses can be with relatively high confidence considered correct, the high computational affinity values should be treated only as preliminary results.

*In silico* analyses were followed with *in vitro* studies which provided further evidence for the inhibitory efficacy of cefazolin as demonstrated by strongly diminished cellular response to IL-2, IL-15, IL-4 and IL-21.

The discovery of the potential binding sites for cefazolin in IL-2/IL-15Rβ and γ_c_ and experimental demonstration of its antagonizing effect towards IL-2-, IL-4-, IL-15- and IL-21- mediated cellular responses presented in this study, strongly substantiates the notion that the antibiotic is a potential inhibitor of all common γ_c_ cytokines. All of the demonstrated effects of cefazolin activity were exhibited at the concentrations similar or lower than plasma concentration observed after intravenous administration of cefazolin accordingly to currently approved dosage instructions^22,23^.

Of interest we find different cefazolin effectiveness in blocking distinct cellular responses. The drug was found more potent in reduction of cytokine dependent IL-17, IFN-γ and TNF-α secretion as compared to less evident (although statistically significant) decrease in cell proliferation. This observation suggests that cefazolin may exert action through differently regulated signal transduction pathways^24^. Also, besides antagonizing the γ_c_ cytokine receptors, additional mechanisms of the drug action, such as for example ligand-induced receptor endocytosis, cannot be excluded. The internalization of the activated cytokine receptors may be a means of signal attenuation, regulation of the duration of receptor signaling or control of signaling output specificity. Studies describing endocytosis of the γ_c_ and IL-2/IL-15Rβ showed that these receptors undergo constitutive internalization followed by lysosomal degradation. On the other hand, IL-2Rα is transported independently of other receptor subunits to recycling endosomes and never reaches lysosomes, which probably contributes to relatively high stability of this subunit. However, it is still not clear whether IL-2R requires endocytosis for signaling. In contrast, ligand-stimulated IL-4R exclusively requires internalization for signal propagation^25^. The above-mentioned mechanisms may, at least in part, explain discrepancies between computationally estimated affinities and *in vitro* data showing inhibitory efficacy of cefazolin lower than predicted.

Certain attention we would like to draw towards IL-2-dependent IL-17 secretion by PBMC revealed in our study. IL-17 release by immune cells following IL-2 is a known phenomenon^26^. Recently, a key role for IL-2 acting in synergy with IL-1β in facilitating IL-17 production has been confirmed in pulmonary γδ T cells^27^. While these observations contrast from the data showing the limiting role of IL-2 in IL-17 production^28,29^, they also stress the necessity to define cefazolin action in more complex context of IL-17-producing helper T cell (Th17 cell) subset. Demonstration of anti-inflammatory effects of cefazolin may expedite possible future drug repositioning. Chemically, the molecule is a derivative of 7-aminocephalosporanic acid and belongs to cephalosporines – semi-synthetic β-lactam antibiotics. They exert antimicrobial activity by binding to the active sites and inhibiting D-alanyl carboxypeptidase and transpeptidase, bacterial enzymes involved in the synthesis of bacterial cell wall. As a result, cephalosporines block structural crosslinking of peptidoglycans (murein) in the bacterial cell walls. To date, the only report on non-antimicrobial anti-inflammatory effect of cephalosporins points towards their ability to inhibit elastase activity^30^. Proteases, and particularly elastase, are important mediators of various inflammation-associated phenomena, such as production of prostaglandins, increased vascular permeability or tissue damage events, therefore blocking the enzyme may reduce inflammation-driven damage.

Results presented in this study suggest that cefazolin activity might be particularly relevant for the development of inhibitors targeting inflammatory signals triggered with IL-2, IL-4, IL-7, IL-9, IL-15 or IL-21. The concept of common γ_c_ cytokines as targets of immunotherapy has been validated experimentally. For example, siRNA targeting the IL-2/IL-15Rβ chain used in adjuvant-induced arthritis in rats was effective in reducing disease severity^31^ and a small-molecule phenylpyrazoleanilide Y-320 inhibited T cell activation induced with IL-15 and reduced type II collagen-induced arthritis in mice and cynomolgus monkeys^32^. JAK inhibitors are considered equally promising as therapeutic agents blocking common γ_c_ cytokines signaling pathways. Following binding to their receptors, all γ_c_ cytokines transmit signal through JAK proteins, which activate signal transducer and activator of transcription (STAT) proteins. Several small-molecule JAK inhibitors of efficacy proven *in vitro* and in animal models of autoimmune diseases, transplant rejection and inflammation are currently evaluated in clinical trials^33^. In 2012, FDA approved Tofacitinib, the first JAK inhibitor for the treatment of rheumatoid arthritis, providing a proof of promising clinical utility of small-molecule therapies targeting cytokine-mediated signaling^34^. Furthermore, pervasive activation of the γ_c_/JAK/STAT system was recently identified in aberrantly proliferating cells in all of the investigated T cell malignancies^35^. For this reason antagonists of cytokine-receptor interactions, and JAK kinase inhibitors are believed to provide a breakthrough in the management of T cell malignancies.

Despite the rapid growth in a number of JAK antagonists, the library of common γ_c_ cytokines small-molecule inhibitors exerting biological activity still remains relatively small. A few selective antagonists of IL-2·IL-2R interaction developed using fragment-based approaches^36^ and computational studies^37^ were proved effective in blocking IL-2 dependent phosphorylation of STAT5, cell proliferation and oxidative burst. Suramin was shown to block binding of IL-4 to its receptors on human tumor cells and IL-4-induced mitogenic response^38^. In a recent study, BNZ-1 – a pegylated peptide designed to specifically bind the γ_c_ receptor and to selectively block IL-2, IL-15, and IL-9 signaling – was demonstrated to inhibit cytokine-mediated cell viability and downstream signaling as well as to increase apoptosis in T-cell large granular lymphocyte leukemia (T-LGLL) cell line and in cells obtained from T-LGLL patients^39^. The collection of newly discovered IL-15 antagonists contains also small-molecule inhibitors of IL-15, IL-15Rα and IL-2/IL-15Rβ identified *in vitro* studies performed by our group^5^ and by others^40^. Their biological effect was demonstrated as suppression of IL-15 dependent IL-17 and TNF- α production^5^ and cell proliferation^40^. It should be noted that γ_c_ family cytokines receptor subunits may exert their regulatory effects not only as membrane receptors but also as secreted proteins. It has been documented that the circulating form of IL-15 is in complex with soluble IL-15Rα (sIL-15Rα). IL-15·sIL-15Rα heterodimer surpasses in stability and activity the poorly secreted and unstable monomeric IL-15^41–43^. Growing evidence points also to soluble γ_c_ (sγ_c_) as a contributor to the aggravation of inflammatory autoimmune diseases^44^. Therefore, both sIL-15Rα and sγ_c_ have been suggested as therapeutic targets for inflammatory diseases.

**Figure 6:**
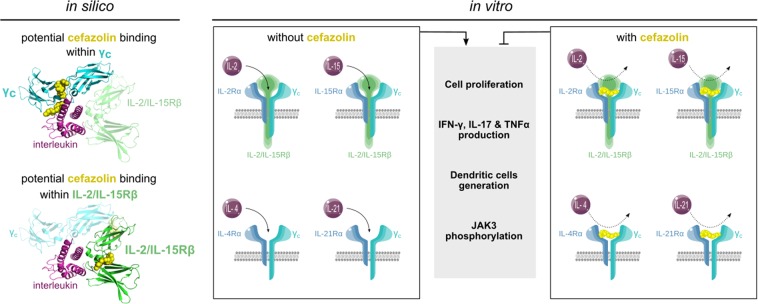
Graphical summary of the mechanism underlying cefazolin effects on IL-2, IL-4, IL-15 and IL-21.

In conclusion, the results of our *in silico* and *in vitro* analyses, summarized graphically in Fig. 6, suggest that cefazolin should be considered as a potential inhibitor of all γ_c_ family cytokines. These novel insights into cefazolin action merit further steps of clinical development necessary for the authorization of the drug use in immunotherapeutic intervention.

## Methods

All methods were carried out in accordance with relevant guidelines and regulations.

### Computational methods

In the computational part of this work we used crystal structures (PDB code: 5M5E^45^) of the cytokine receptor subunits gamma and beta. We have removed water molecules from both receptor subunits and added hydrogen atoms to these structures with AutoDockTools 4^46^. Atomic interaction energy grids have been calculated using probes corresponding to each atomic type found in the cefazolin, at 0.375 Å grid resolution. In all docking experiments cefazolin has been treated in a fully flexible manner with the Gasteiger partial charges added by AutoDockTools 4. For both, beta and gamma subunits, a two-step docking protocol was used to assess the most likely binding site of cefazolin to the beta and gamma subunits. Due to the large size of IL-2 receptor, in the first step two 126 Å cubic boxes that spanned over the entire beta subunit as well as two 126 Å cubic boxes spanning over the entire gamma subunit were used and all amino acids were treated as rigid. We used Autodock 4.2^46^ with the Genetic Lamarckian Algorithm and standard options, but including 1,000 dockings per compound and 5,000,000 energy evaluations per docking^47^. Such an approach allowed us to identify six potential binding sites within the gamma subunit and six within the beta subunit with relatively low estimates of cefazolin binding affinities (<700 nM). In the second step more accurate docking to these potential sites was performed using the same docking method but treating selected amino acids as flexible ones (see Table 1).

**Table 1:** List of flexible residues in the twelve potential binding sites investigated using flexible docking.

Potential binding site	List of flexible residues
IL-2/IL-15Rβ 1	Gln113, Val114, His120, Arg204, Lys206
IL-2/IL-15Rβ 2	Ser147, His150, Glu154, Glu170, Thr171
IL-2/IL-15Rβ 3	Cys10, Phe11, Gln96, Lys99, Asn103, Phe191
IL-2/IL-15Rβ 4	Asn13, Asn17, Ser19, Asn61, Arg105, Gln130
IL-2/IL-15Rβ 5	Ser6, Gln7, Trp22, Gln96, Asn103
IL-2/IL-15Rβ 6	Arg105, Met107, Ile110, Ser111, Gln199
γ_c_ 1	Gln39, Gln122, Lys125, Asn128, Gln213, His214
γ_c_ 2	Lys70, Asn71, Tyr103, Lys125, Gln127, Asn128
γ_c_ 3	Leu35, Lys70, Gln119, Thr121
γ_c_ 4	Trp68, Lys70, Asn71, Asp75, Val77
γ_c_ 5	Asn44, Val45, Lys97, Tyr103, Arg155, Phe156
γ_c_ 6	Asn137, Leu138, Leu140, His220, His223

### Cefazolin preparation

Cefazolin was purchased from Polpharma (Poland). For all experiments cefazolin 100 mM stock solution was prepared in sterile distilled water. Further dilutions were prepared in culture media.

### Cell isolation and culture

Peripheral blood mononuclear cells (PBMC) were isolated from buffy coats of healthy male donors aged 25–35 years (Warsaw Blood Donation Centre). Thirty ml of blood twice diluted in 0.9% NaCl was layered on 15 ml of Lymphoprep (Axis-shield) and centrifuged at 800 x g for 15 minutes. The layer of PBMC was collected, the cells were washed twice in 0.9% NaCl and suspended in RPMI 1640 medium (Gibco), containing 10 mM HEPES (Sigma-Aldrich), 10% fetal bovine serum (FBS) (Biowest) and 1% antibiotic-antimycotic (AA) solution (streptomycin sulfate, sodium penicilate G, amphotericin B) (PAA) and seeded for the experiment.

NK cells were isolated from 1 × 10^8^ freshly isolated PBMC using Human NK Cell Enrichment Kit (Stemcell Technologies) using the Purple Easysep Magnet (Stemcell Technologies) according to the manufacturer’s instructions. Isolated cells were suspended in RPMI 1640 containing 10 mM HEPES, 10% FBS and 1% AA solution and seeded for the experiment.

TF-1 cells (ACC 334) were purchased from Deutsche Sammlung von Mikroorganismen und Zellkulturen (DSMZ). Cells were cultured in RPMI 1640 supplemented with 20% FBS, 10 mM HEPES, 2 ng/ml (20 U/ml) granulocyte-macrophage colony stimulating factor (GM-CSF, PeproTech) and 1% AA solution.

NK-92 cells (ACC 4884) were purchased from DSMZ. Cells were cultured in X-vivo 20 (Lonza) supplemented with 5% Human Serum (Sigma-Aldrich) and 1% AA solution.

### MTT cytotoxicity assay

The cytotoxicity of cefazolin for TF-1 and NK cells was assessed using the MTT test. Cells were incubated with cefazolin in concentrations 100, 200, 400 and 800 µM in RPMI 1640 supplemented with 10% FBS and 1% AA solution on 96-well V-bottom plate in 37 °C, 5% CO_2._ Each experimental variant was performed in five replicates. After 48 hours cells were centrifuged (500 x g, 10 minutes) and the medium was discarded. 100 µl per well of 3-(4, 5-dimethylthiazol-2-yl)-2, 5-diphenyltetrazolium bromide (MTT) (Sigma-Aldrich) (1 mg/ml) dissolved in RPMI 1640 medium was added to each well for 1 hour. Then, cells were centrifuged (1,500 x g, 10 minutes) and the medium was discarded. Formazan crystals were dissolved in 100 µl per well of 0.04 M HCl (Sigma-Aldrich) dissolved in isopropanol (Sigma-Aldrich). Solutions were transferred on flat bottom 96-well plate and the absorbance, which is proportional to a number of living cells, was measured at 570 nm with correction wavelength at 680 nm. The experiment was performed using cells isolated from three blood donors, each in five technical replicates. The cytotoxicity of cefazolin for PBMC we have assessed previously^5^.

### BrdU cell proliferation assays

The proliferation of PBMC and TF-1 was evaluated using BrdU Cell Proliferation Assay (Calbiochem).

The freshly isolated PBMC were seeded in a 96-well V-bottom plate (25 × 10^3^ cells in 200 µl of the culture medium/well) and cultured for 24 hours. Next, the cells were incubated for 30 minutes with cefazolin at concentrations of 100 µM, 200 µM and 400 µM. Next, 20 ng/ml IL-2 or 5 ng/ml IL-15 was added and the cells were incubated for 72 hours in 37 °C, 5% CO_2_. For the last 24 hours of incubation bromodeoxyuridine (BrdU) was added to the culture medium at the concentration recommended by the manufacturer. Then, the cells were centrifuged (160 x g, 10 minutes) and fixed. Further experimental steps were performed according to the manufacturer’s protocol. The experiment was performed using cells isolated from three blood donors, each in five technical replicates.

TF-1 cells were starved in a RPMI 1640 with 10% FBS and 1% AA solution for 24 hours. Then 40 × 10^3^ cells were seeded in a 96-well V-bottom plate with or without cefazolin at concentrations of 100 µM, 200 µM and 400 µM and incubated for 30 minutes in 150 µl of RPMI 1640 medium containing 10% FBS and 1% AA solution. Next, 10 ng/ml IL-4 (Invitrogen, Gibco) was added and the cells were incubated for 72 hours in 37 °C, 5% CO_2_. For the last 24 hours of incubation BrdU was added to the culture medium at the concentration recommended by the manufacturer. Then, the cells were centrifuged (1,000 x g, 10 minutes) and fixed. Further experimental steps were performed according to the manufacturer’s protocol. The experiment was performed three times, each in five technical replicates.

The description of two additional proliferation assays, CFSE cell proliferation assay for PBMC treated with IL-2 and IL-15 and MTT cell cytotoxicity assay for TF-1 stimulated with IL-4, is provided in Supplementary Material and Methods.

### IL-17, TNF-α and IFN-γ secretion

Freshly isolated PBMC were seeded in a 48-well plate (1 × 10^6^ cells in 0.5 ml of the culture medium per well) and treated with cefazolin at 100 µM, 200 µM and 400 µM concentrations for 30 minutes and then 20 ng/ml (420 U/ml) of IL-2, 5 ng/ml (2,250 U/ml) of IL-15 or 5 ng/ml (100 U/ml) of IFN-γ (R&D Systems, Minneapolis, MN, USA) was added for 48 hours. Next, the culture media from each well were collected, centrifuged (10,000 × g, 10 minutes, 4 °C) and frozen at −80 °C until the level of cytokine was measured using ELISA tests. The cells were harvested, lysed in 0.1 M NaOH (Sigma-Aldrich) and frozen at −80 °C until the total protein level measurement. Each experiment was performed using cells isolated from four blood donors, each in two technical replicates.

Freshly isolated NK cells were seeded in a 96-well plate (3 × 10^5^ cells in 150 µl of the culture medium per well) and treated with cefazolin at 100 µM, 200 µM and 400 µM concentrations for 30 minutes and then 5 ng/ml (2,250 U/ml) of IL-15 was added for 48 hours. Next, the culture media from each well were collected, centrifuged (10,000 x g, 10 minutes, 4 °C) and frozen at −80 °C until the measurement of cytokines level. The cells were harvested, lysed in 0.1 M NaOH and frozen at −80 °C until the total protein level measurement. Each experiment was performed using cells isolated from three blood donors, each in two technical replicates.

Total protein levels were measured in PBMC and NK cell lysates using BCA Protein Assay Kit (Pierce Biotechnology). The concentrations of IL-17, IFN-γ and TNF-α were measured in culture supernatants of cytokine-stimulated cells using the Human IL-17A ELISA Kit (Diaclone), Human IFN-γ ELISA Kit (Dicalone) and Human TNF ELISA Kit (BD Biosciences Pharmingen), respectively, according to the manufacturers’ instructions. The values obtained for IL-17, IFN-γ and TNF-α concentrations were calculated per 1 mg of total protein.

### Generation of human monocyte derived DC

DC were obtained through differentiation of human peripheral monocytes *in vitro*. Monocytes were isolated from freshly isolated PBMC using EasySep™ Human CD14 Positive Selection Kit II (StemCell Technologies) and the Purple Easysep Magnet (Stemcell Technologies) according to the manufacturer’s instructions. Isolated monocytes were suspended in AIM-V (Gibco/Invitrogen) culture medium supplemented with recombinant human GM-CSF (13.3 ng/ml; 200 U/ml; R&D Systems) and recombinant human IL-4 (17.2 ng/ml; 500 U/ml; R&D Systems). Cells were incubated for 5 days in 37 °C, 5% CO_2_ with or without 100, 200 or 400 µM cefazolin. Then, cells were collected and washed twice with PBS (600 x g, 8 minutes). Differentiation of monocytes into DC was evaluated by flow cytometry, using the fluorochrome-conjugated monoclonal antibodies: CD11c-APC and CD14-PE (both from eBioscience). Cells were incubated with appropriate monoclonal antibodies for 30 minutes at 4 °C, then washed twice in CellWash buffer (BD Biosciences, Franklin Lakes, NJ, USA) and analysed by flow cytometry (FACSVerse, BD Biosciences) using FACSuite software (BD Biosciences). Fluorescence minus one (FMO) controls were performed and compensation was adjusted using BD CaliBRITE Beads (BD Biosciences). CD11c+ CD14− cells were identified as DC. Surface expression of CD11c was estimated as mean fluorescence intensity (MFI). Cells viability was measured using a Muse Count and Viability Assay kit (Merck Millipore) followed by a Muse Cell Analyzer (Merck Millipore) according to the manufacturer’s instructions.

### Western blot analysis

Freshly isolated PBMC (20 × 10^6^) were incubated for 30 minutes at 37 °C in culture medium with or without cefazolin (100 µM, 200 µM or 400 µM). Next, cells (5 × 10^6^ in each variant) were incubated for 10 minutes at 37 °C with or without IL-2 (20 ng/ml; 420 U/ml), IL-4 (10 ng/ml; 290/U/ml) or IL-15 (5 ng/ml; 2,250 U/ml).

TF-1 cells were starved in RPMI 1640 supplemented with 2% FBS and 1% AA solution for 24 hours. Then, 6 × 10^6^ cells were incubated for 30 minutes at 37 °C in culture medium with or without cefazolin (100 µM, 200 µM and 400 µM). Next, 3 × 10^6^ cells were incubated for 10 minutes at 37 °C with or without IL-4 (10 ng/ml; 290 U/ml).

NK-92 cells were starved in RPMI 1640 supplemented with 10% FBS and 1% AA solution for 24 hours. Then, 6 × 10^6^ cells were incubated for 30 minutes 37 °C in culture medium with or without cefazolin (100 µM, 200 µM or 400 µM). Next, 3 × 10^6^ cells were incubated for 10 minutes at 37 °C with or without IL-21 (100 ng/ml).

After incubation all types of cells were centrifuged (1,000 x g, 10 minutes, 4 °C), washed with ice-cold 0.9% saline and lysed in 30 µl of cOmplete Lysis M with PhosSTOP buffer (Roche) for 10 minutes at room temperature. Then the lysates were centrifuged (14,000 x g, 15 minutes, 4 °C), supernatants were transferred to fresh microcentrifuge tubes and total protein levels were measured using BCA Protein Assay Kit (Pierce Biotechnology). Lysates were frozen at −80 °C for further use.

Cell lysates containing 50 µg of total protein were mixed with Laemmli buffer, boiled for 5 minutes, electrophoresed on 10% sodium dodecyl sulfate-polyacrylamide gel and transferred to polyvinylidene fluoride (PVDF) membranes. The blots were blocked with 5% nonfat dry milk in Tris-Buffered Saline containing 0.1% Tween-20 (TBST) for 1 hour at room temperature. Membranes were then probed overnight at 4 °C with primary rabbit monoclonal anti-human phospho-JAK3 antibody (1:1,000, #5031, Cell Signaling Technologies) dissolved in 5% bovine serum albumin (BSA) in TBST. Immunodetection of specific proteins was carried out with horseradish peroxidase-conjugated goat anti-rabbit IgG (1:1,000, #7074, Cell Signaling Technologies), using the enhanced chemiluminescence (ECL) system (WesternBright ECL, Advansta) according to the manufacturer’s instructions. Next, blots were stripped with Restore PLUS Western Blot Stripping Buffer (Pierce Biotechnology) for 10 minutes at room temperature, blocked with 5% nonfat dry milk in TBST for 1 hour at room temperature and probed overnight at 4 °C with rabbit monoclonal anti-human JAK3 antibody (1:3,000, #8827, Cell Signaling Technologies) dissolved in 5% BSA in TBST. After detection blots were stripped again, blocked with 5% nonfat dry milk in TBST for 1 hour at room temperature and probed for one hour at room temperature with mouse monoclonal anti-β-actin antibody conjugated with peroxidase (1:100,000, #A3854, Sigma-Aldrich) dissolved in 5% nonfat dry milk in TBST. Every experiment was performed at least three times.

### Statistical analysis

Statistical significance was assessed by ANOVA with Dunnet post hoc test. P values below 0.05 were considered statistically significant. Statistical analyses were performed using GraphPad Prism 5 software (GraphPad Software). Data were presented as the mean ± SEM from at least three independent experiments.

## Supplementary information

Supplementary information.
