# Supplementary material for: The anti-inflammatory potential of cefazolin as common gamma chain cytokine inhibitor
Source: Sci Rep. 2020 Feb 19;10:2886. doi: 10.1038/s41598-020-59798-3 (PMC7031511; doi:10.1038/s41598-020-59798-3)
Supplement: Supplementary file 1 — Supplementary information. [file 41598_2020_59798_MOESM1_ESM.pdf]

The anti-inflammatory potential of cefazolin as common gamma chain cytokine inhibitor

Barbara Żyżyńska-Granica [1,2], Bartosz Trzaskowski [3], Małgorzata Dutkiewicz [4], Oliwia Zegrocka-Stendel [4], Maja Machcińska [5,6], Katarzyna Bocian [6], Magdalena Kowalewska [4,7] and Katarzyna Koziak [4,*]

[1] Department of Pharmacodynamics, Centre for Preclinical Research and Technologies, Medical University of Warsaw, Banacha 1b, 02-097, Warsaw, Poland; barbara.zyzynska@wum.edu.pl

[2] Chair and Department of Biochemistry, Medical University of Warsaw, Banacha 1, 02-097 Warsaw, Poland

[3] University of Warsaw, Centre of New Technologies, Banacha 2c, 02-097 Warsaw, Poland; b.trzaskowski@cent.uw.edu.pl

[4] Department of Immunology, Biochemistry and Nutrition, Centre for Preclinical Research and Technologies, Medical University of Warsaw, Banacha 1b, 02-097, Warsaw, Poland; e-mails: mdutkiewicz@wum.edu.pl, ostendel@wum.edu.pl, mkowalewska@wum.edu.pl, katarzyna.koziak@wum.edu.pl

[5] Laboratory of Parasitology, Military Institute of Hygiene and Epidemiology, Kozielska 4, 01-163 Warsaw, Poland; maja.machcinska@wihe.pl

[6] Department of Immunology, Faculty of Biology, University of Warsaw, Miecznikowa 1, 02-096 Warsaw, Poland; kbocian@biol.uw.edu.pl

[7] Department of Molecular and Translational Oncology, Maria Sklodowska-Curie Institute - Oncology Centre, Roentgena 5, 02-781, Warsaw, Poland

[*] Correspondence: katarzyna.koziak@wum.edu.pl; tel.: +48-22-116-6128

Supplementary Methods and Figures

CFSE cell proliferation assay

Freshly isolated PBMC were seeded in RPMI 1640 supplemented with 10% FBS, 10 mM HEPES and 1% AA solution and stimulated with 1µg/ml PHA for 48 hours. Next, the cells were centrifuged (300 x g, 8 minutes) and starved for 6 hours in RPMI 1640 supplemented with 1% FBS. Then, cells were centrifuged, resuspended in prewarmed (37°C) PBS with 1% FBS and incubated with CellTrace™ CFSE (CellTrace™ CFSE Cell Proliferation Kit, Invitrogen) according to the manufacturer's protocol. Stained cells were centrifuged, resuspended in prewarmed medium and 100 µM, 200 µM and 400 µM cefazolin was added. After 30 minutes 20 ng/ml IL-2 (PeproTech) and 5 ng/ml IL-15 (R&D Systems) were added and cells were incubated for 5 days in 37°C, 5% CO_2. Next, the cells were centrifuged (500 x g, 8 minutes), resuspended in PBS and 10,000 cells per sample were analysed by flow cytometry on a FACSCalibur using CellQuest software (Beckton Dickinson). The experiment was repeated using cells isolated from four different blood donors.

MTT cell cytotoxicity assay

TF-1 cells were starved in a RPMI 1640 with 10% FBS and AA solution, without GM-CSF for 24 hours. Then 4 x 10^4 cells were seeded in a 96-well V-bottom plate with or without cefazolin at concentrations of 100 µM, 200 µM and 400 µM and incubated for 30 minutes in 150 µl of RPMI 1640 medium containing 10% FBS and 1% AA solution. Next, 10 ng/ml IL-4 was added and the cells were incubated for 72 hours in 37°C, 5% CO_2. Then, the plate was centrifuged (500 x g, 10 minutes) and 90 µl of 1 mg/ml MTT in RPMI 1640 was added to each well. After 2 hour incubation the plate was centrifuged (1,500 x g, 10 minutes), the medium was discarded and 100 µl per well of 0.04 M HCl suspended in isopropanol was added. Then, 60 µl of the solution containing dissolved formazan crystals was transferred to a new flat-bottom plate and the absorbance was read at 570 nm with the reference wavelength of 680 nm. The experiment was performed four times, each in five technical replicates.

Supplementary Figures

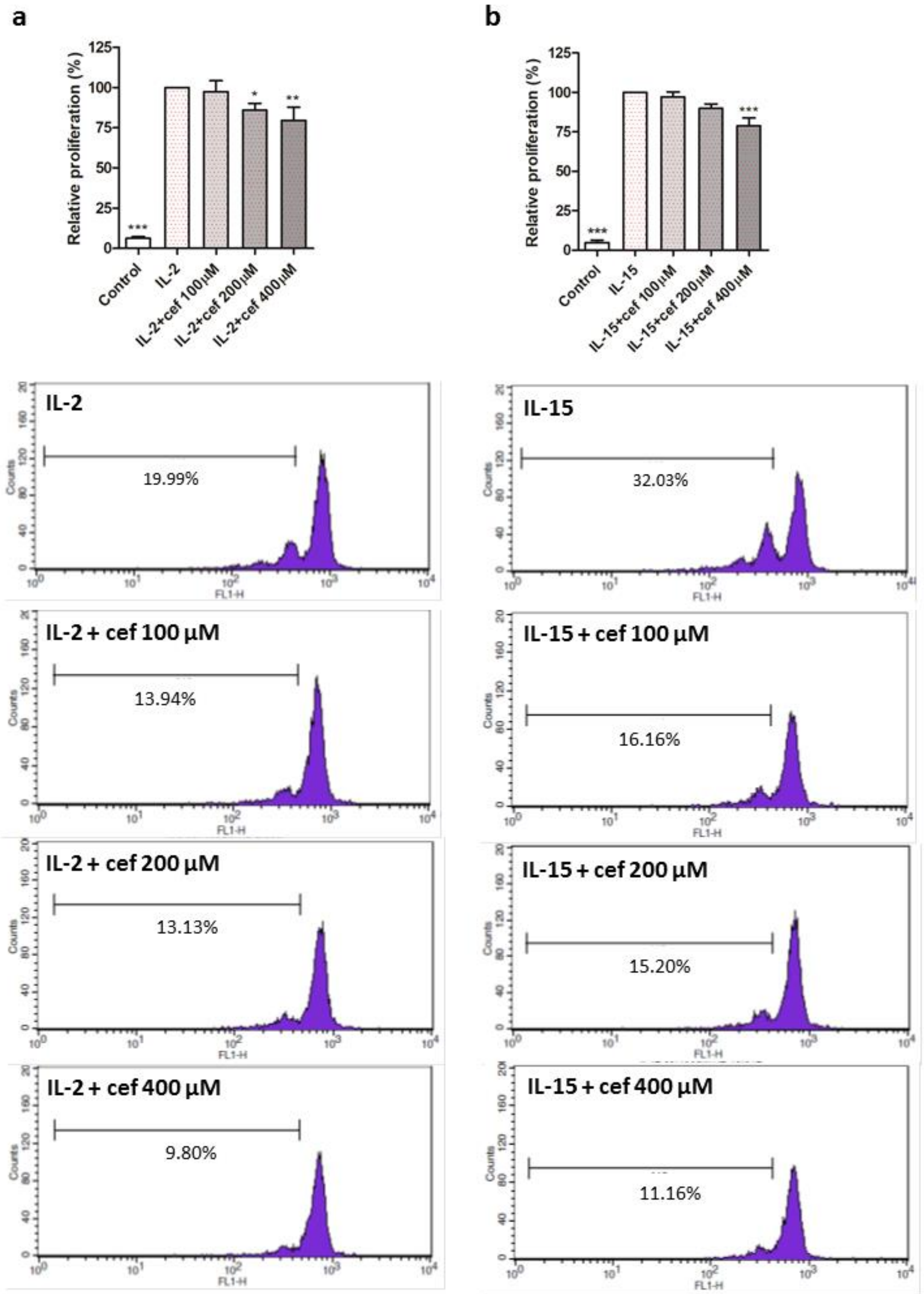

Figure S1. The influence of cefazolin on IL-2-(a) and IL-15-induced (b) PBMC proliferation measured by CFSE assay using flow cytometry method. The results are presented as the percentage of cell response compared to IL-2- (**a**) and IL-15- (**b**) treated cells that were defined as 100%. Control refers to unstimulated cells. The results from three independent experiments ($n = 3$) are presented as mean ± SD. Statistical significance was assessed by ANOVA with Dunnet post hoc test. *$p < 0.05$, **$p < 0.01$, ***$p < 0.001$. Representative histograms for CFSE-stained cells are presented in the lower panel.

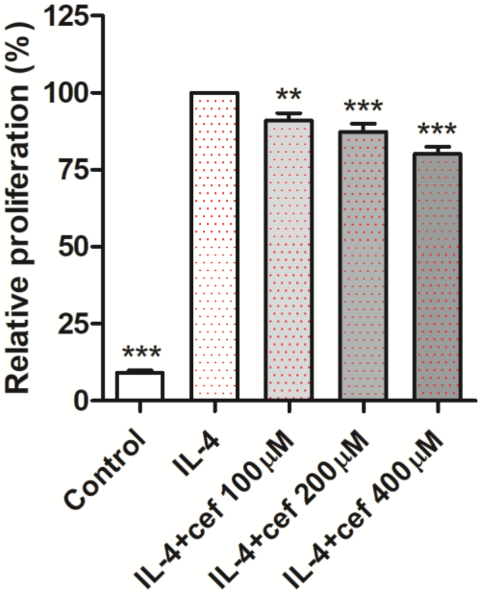

Figure S2. The influence of cefazolin on IL-4-induced TF-1 proliferation measured by MTT assay. The results are presented as the percentage of cell response compared to IL-4 treated cells that was defined as 100%. Control refers to unstimulated cells. The results from three independent experiments ($n = 3$) are presented as mean ± SD. Statistical significance was assessed by ANOVA with Dunnet post hoc test. *$p < 0.05$, **$p < 0.01$, ***$p < 0.001$.

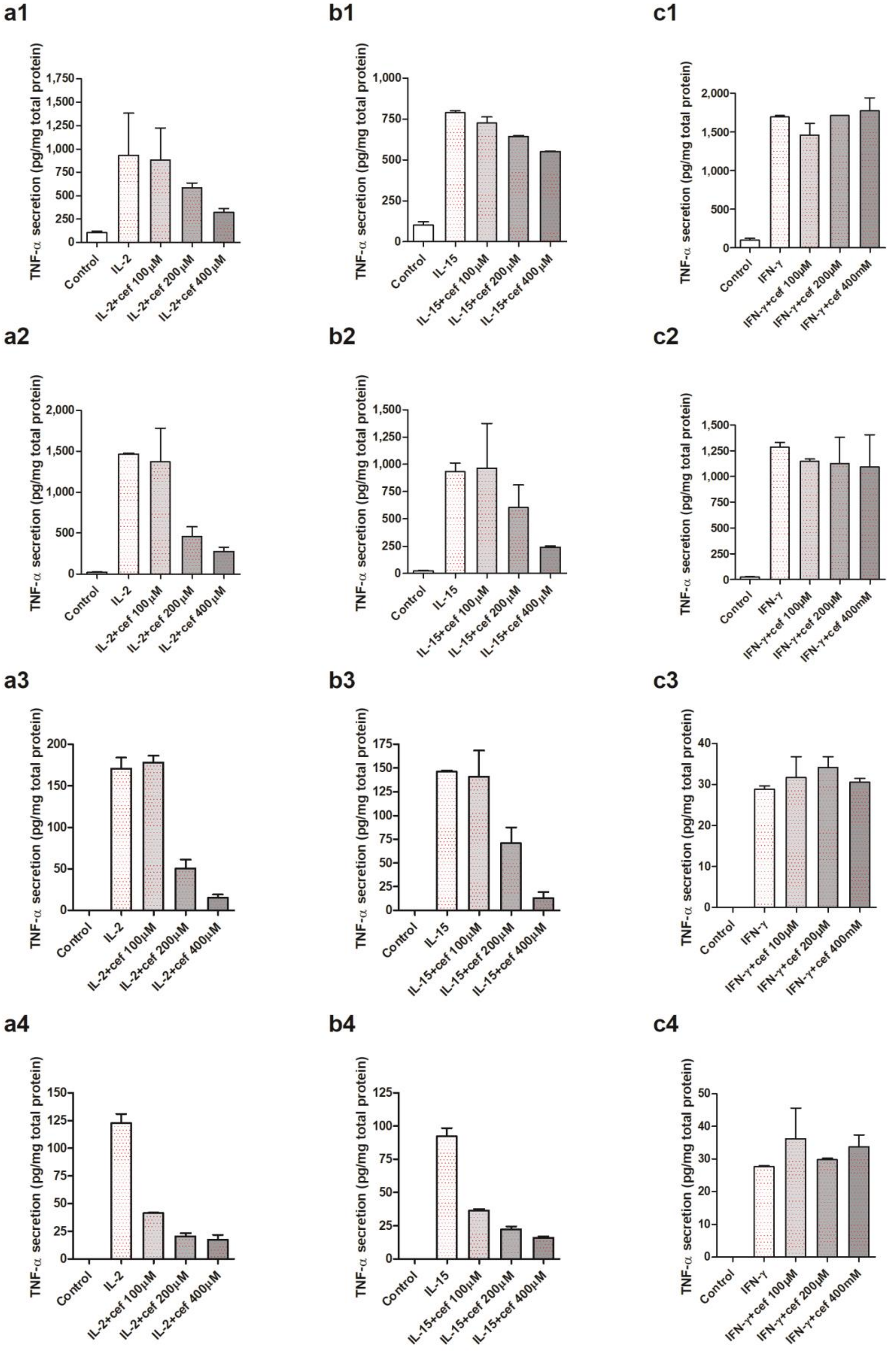

Figure S3. The influence of cefazolin on TNF-α release – single donor data. TNF-α secretion was measured in IL-2 (**a1-a4**), IL-15 (**b1-b4**) and IFN-γ (**c1-c4**) stimulated PBMC. The results are presented in pg/mg of total protein. The data obtained for single donors is shown in panels **a1**, **b1**and **c1** for donor 1; **a2**, **b2** and **c2** for donor 2; **a3**, **b3** and **c3** for donor 3; **a4**, **b4** and **c4** for donor 4.

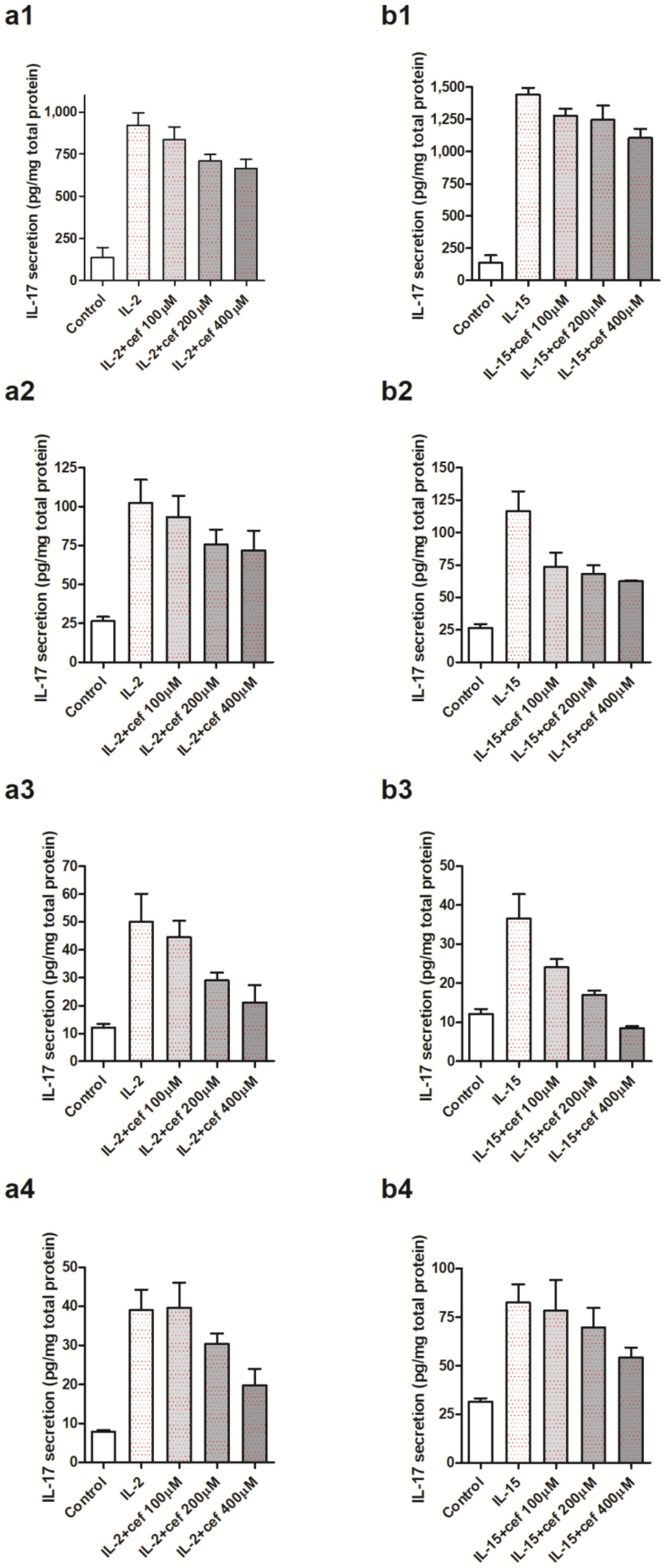

Figure S4. The influence of cefazolin on IL-17 release – single donor data. IL-17 secretion was measured in IL-2 (**a1-a4**) and IL-15 (**b1-b4**) stimulated PBMC. The results are presented in pg/mg of total protein. The data obtained for single donors is shown in panels **a1** and **b1** for donor 1; **a2** and **b2** for donor 2; **a3** and **b3** for donor 3; **a4** and **b4** for donor 4.

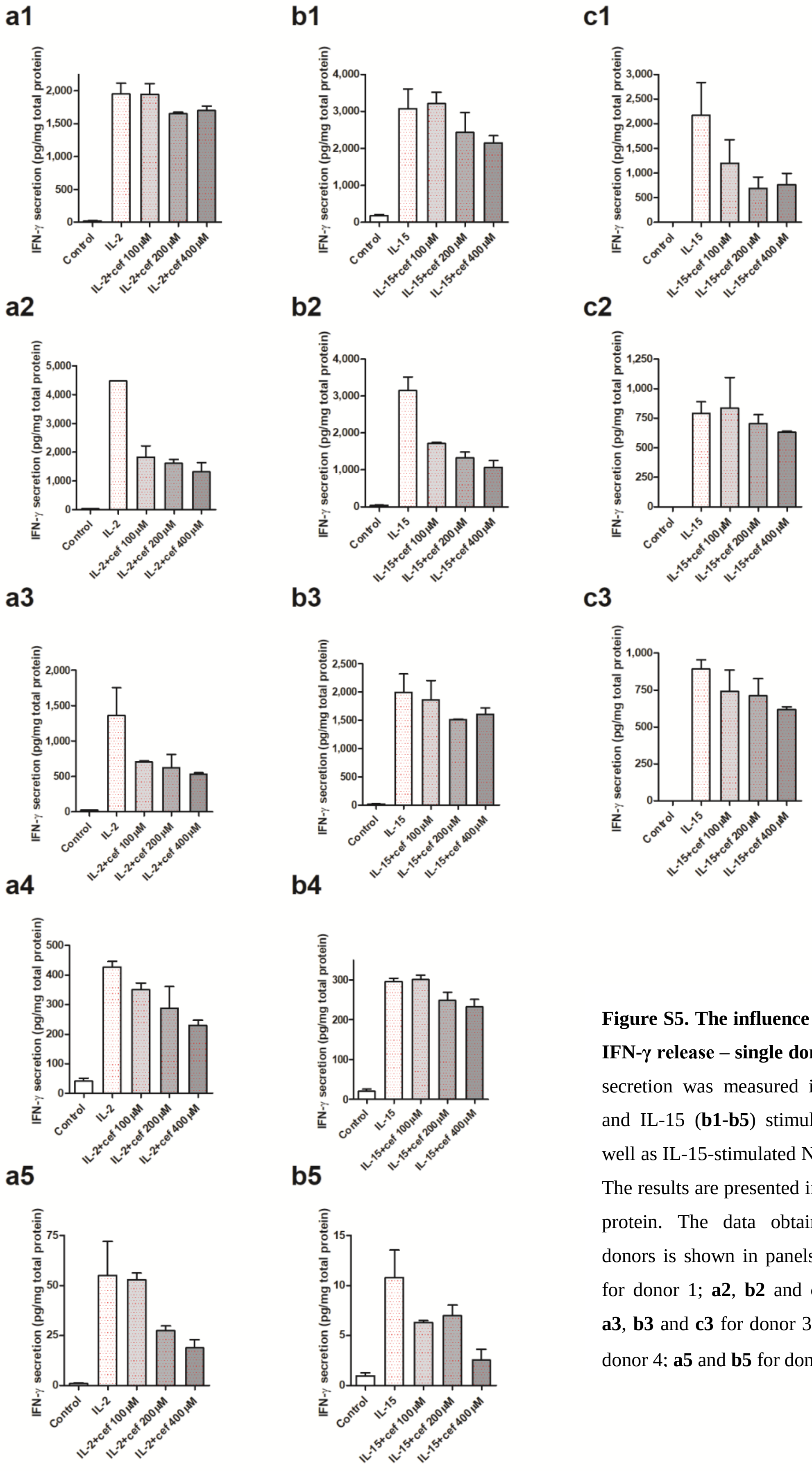

Figure S5. The influence of cefazolin on IFN-γ release – single donor data. IFN-γ secretion was measured in IL-2 (**a1**-**a5**) and IL-15 (**b1**-**b5**) stimulated PBMC as well as IL-15-stimulated NK cells (**c1**-**c3**). The results are presented in pg/mg of total protein. The data obtained for single donors is shown in panels **a1**, **b1** and **c1** for donor 1; **a2**, **b2** and **c2** for donor 2; **a3**, **b3** and **c3** for donor 3; **a4 and b4** for donor 4; **a5** and **b5** for donor 5**.**

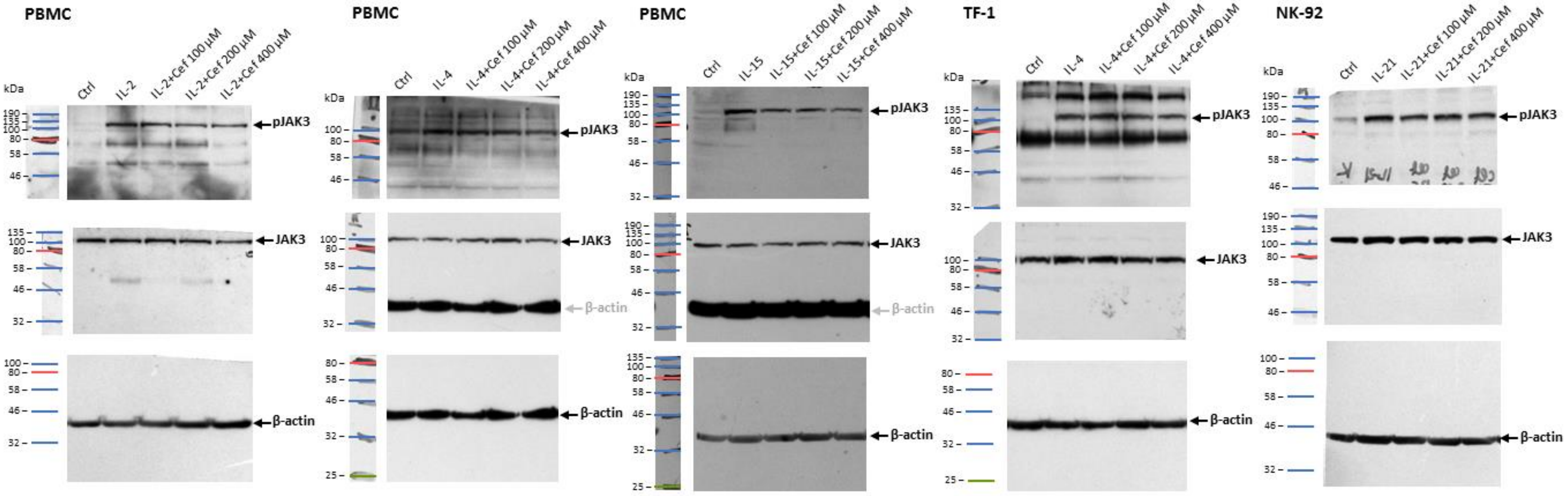

Figure S6. **Full length representative blots from Figure 5 showing cefazolin effect on JAK3 phosphorylation**. Western blots for phospho-JAK3 (pJAK3), total JAK3 (JAK3) and β-actin in cell lysates were obtained after cytokine and cefazolin treatment of: PBMC stimulated with IL-2; PBMC stimulated with IL-4; PBMC stimulated with IL-15; TF-1 cells stimulated with IL-4; NK-92 cells stimulated with IL-21.

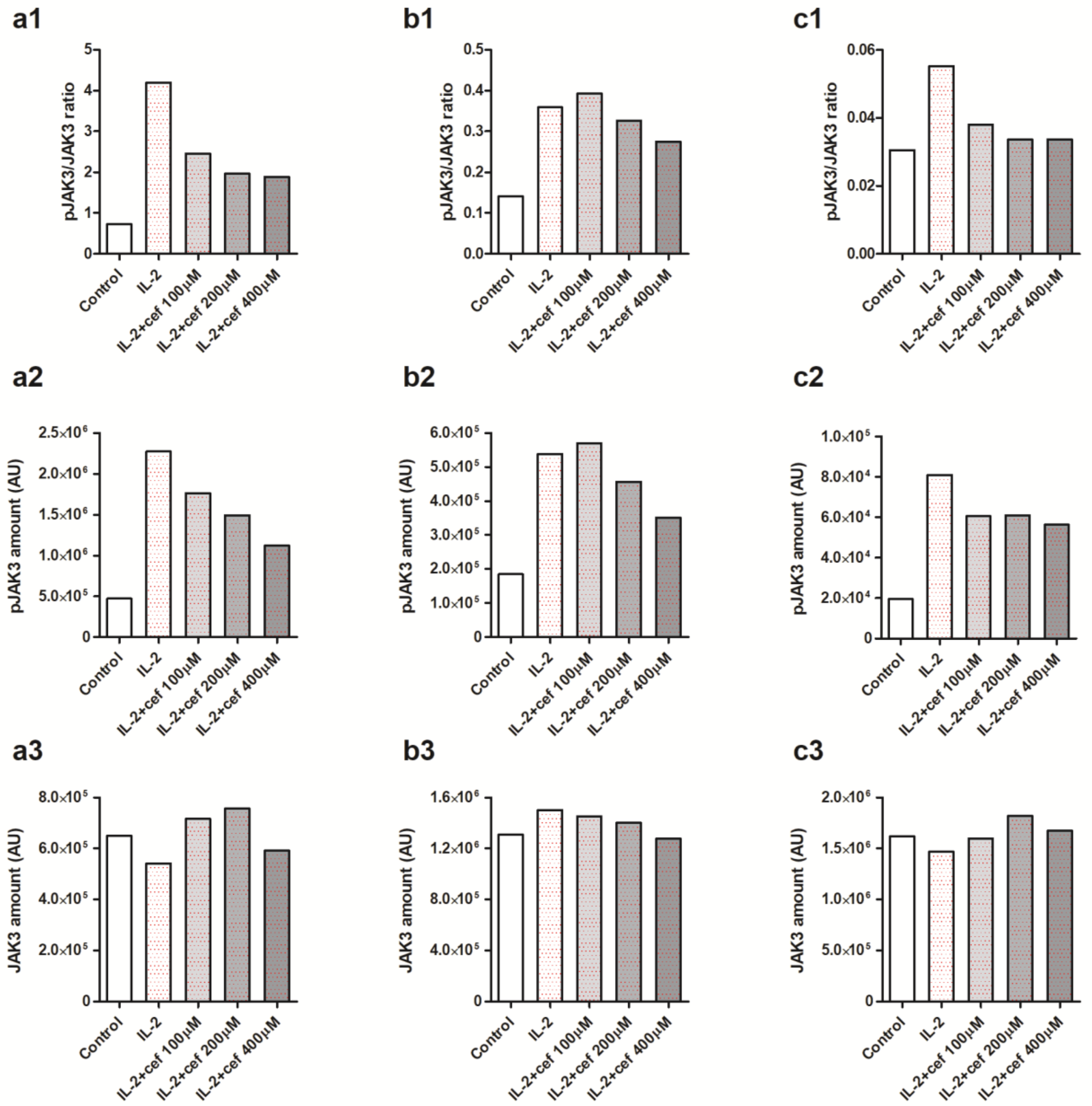

Figure S7. Cefazolin effect on JAK3 phosphorylation in PBMC stimulated with IL-2 – single donor data. Western blot densitometry values (AU) for JAK3 and phospho-JAK3 (pJAK3) for single donors are presented in **a3**, **b3**, **c3** and in **a2**, **b2**, **c2**, respectively. Panels **a1**, **b1** and **c1** depict changes in pJAK3/JAK3 ratio calculated for each donor.

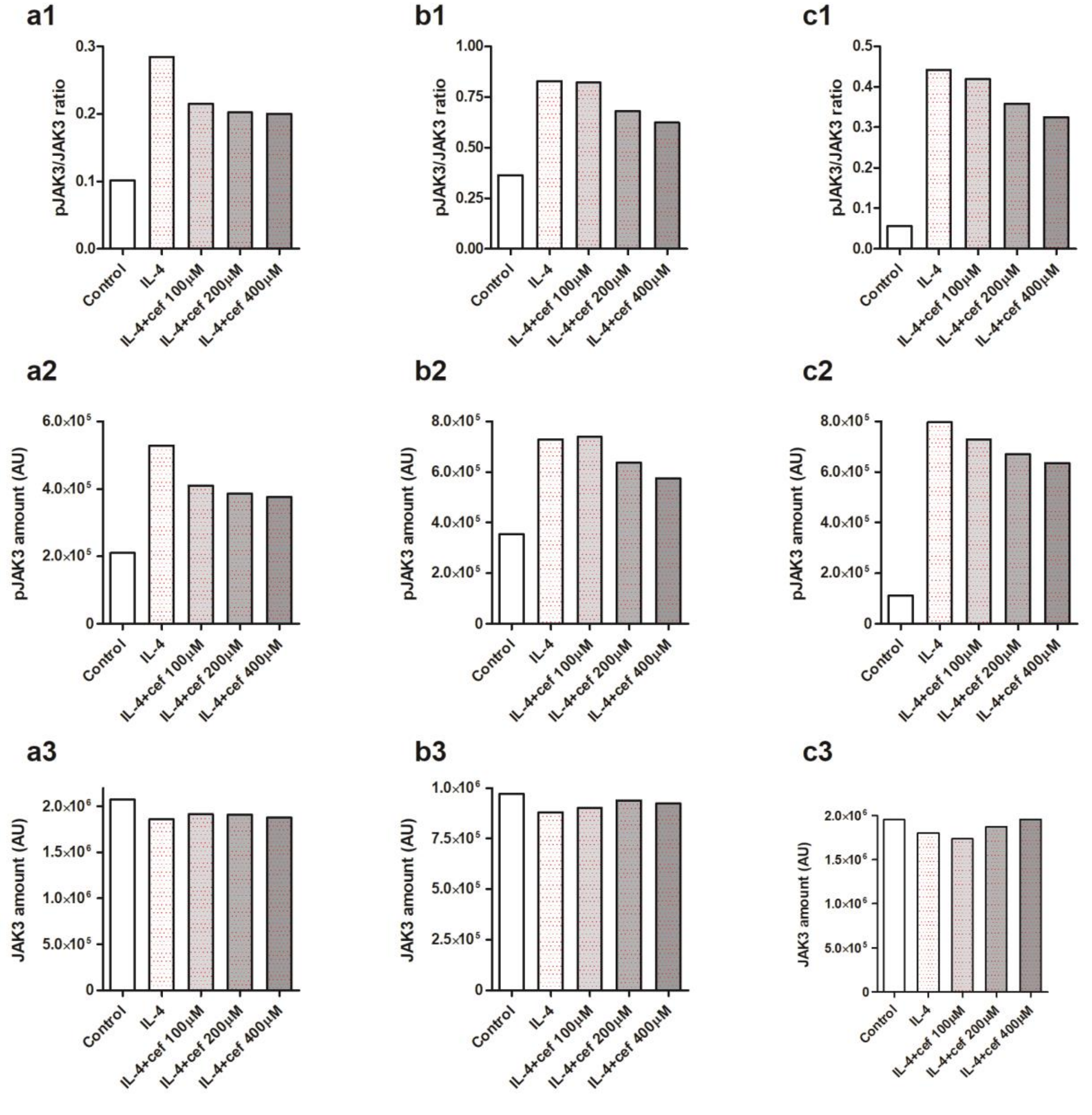

Figure S8. Cefazolin effect on JAK3 phosphorylation in PBMC stimulated with IL-4 – single donor data. Western blot densitometry values (AU) for JAK3 and phospho-JAK3 (pJAK3) for single donors are presented in **a3**, **b3**, **c3** and in **a2**, **b2**, **c2**, respectively. Panels **a1**, **b1** and **c1** depict changes in pJAK3/JAK3 ratio calculated for each donor.

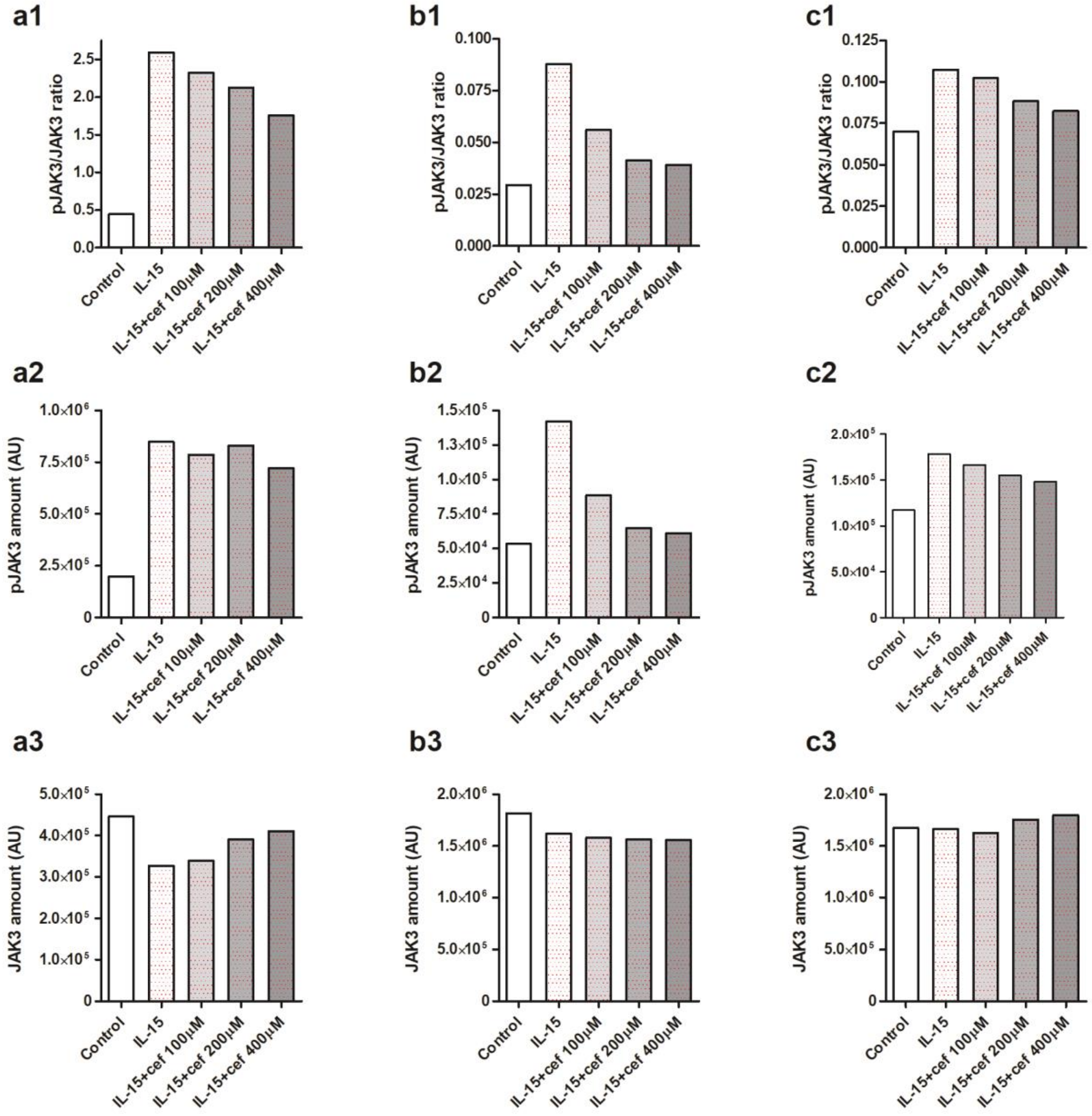

Figure S9. Cefazolin effect on JAK3 phosphorylation in PBMC stimulated with IL-15 – single donor data. Western blot densitometry values (AU) for JAK3 and phospho-JAK3 (pJAK3) for single donors are presented in **a3**, **b3**, **c3** and in **a2**, **b2**, **c2**, respectively. Panels **a1**, **b1** and **c1** depict changes in pJAK3/JAK3 ratio calculated for each donor.

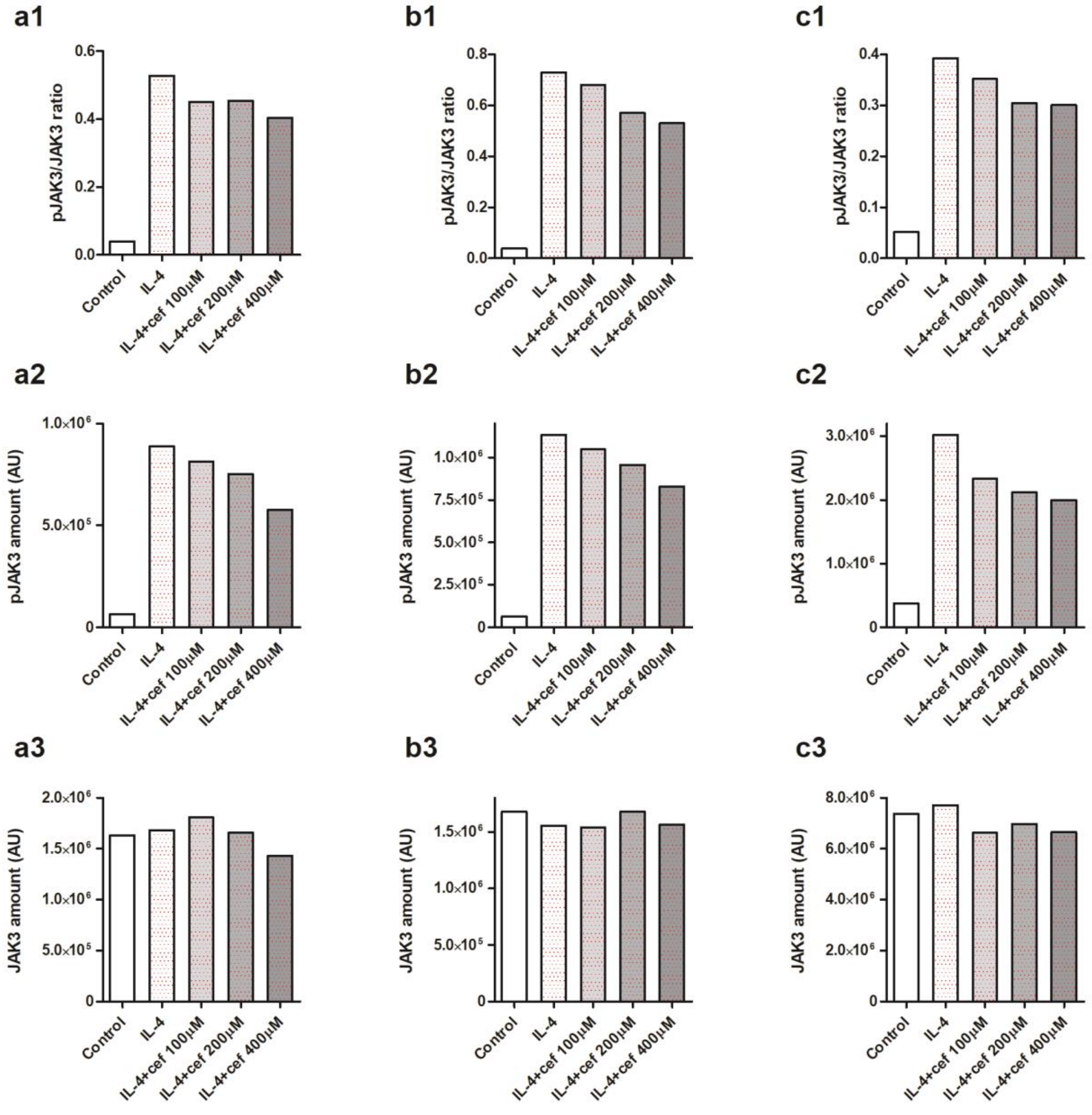

Figure S10. Cefazolin effect on JAK3 phosphorylation in TF-1 cells stimulated with IL-4 – single donor data. Western blot densitometry values (AU) for JAK3 and phospho-JAK3 (pJAK3) for single donors are presented in **a3**, **b3**, **c3** and in **a2**, **b2**, **c2**, respectively. Panels **a1**, **b1** and **c1** depict changes in pJAK3/JAK3 ratio calculated for each donor.

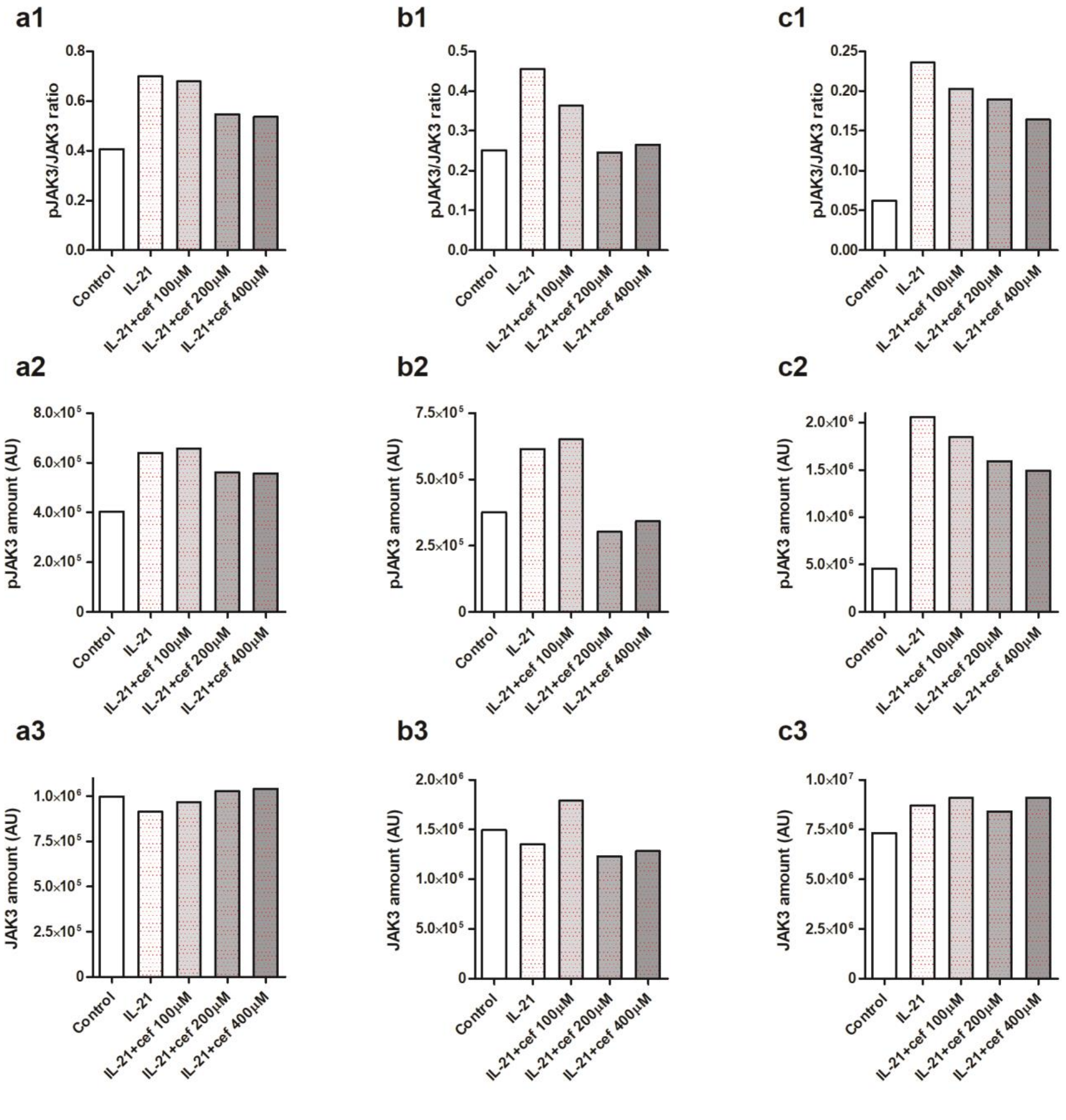

Figure S11. Cefazolin effect on JAK3 phosphorylation in NK-92 cells stimulated with IL-21 – single donor data. Western blot densitometry values (AU) for JAK3 and phospho-JAK3 (pJAK3) for single donors are presented in **a3**, **b3**, **c3** and in **a2**, **b2**, **c2**, respectively. Panels **a1**, **b1** and **c1** depict changes in pJAK3/JAK3 ratio calculated for each donor.
